# Computational exploration of the binding mode of the heme-dependent activator YC-1 into the active catalytic site of soluble guanylate cyclase

**DOI:** 10.1186/2050-6511-16-S1-A32

**Published:** 2015-09-02

**Authors:** Luis Agulló, Ignasi Buch, Hugo Gutiérrez de Terán, Gianni de Fabritis, David Garcia-Dorado, Jordi Villà-Freixa

**Affiliations:** 1 Computational Biochemistry and Biophysics Laboratory (CBBL), U_Science Tech (UST), Universitat de Vic - Universitat Central de Catalunya (UVic-UCC), 08500 Vic, Barcelona, Spain; 2 Research Program on Biomedical Informatics (GRIB), IMIM, Barcelona, Spain; 3 Dept. Cell & Mol. Biol., Uppsala Biomedicinska Centrum BMC, Husarg. 3, 75124 Uppsala, Sweden; 4 Lab. Experimental Cardiology, Vall d’Hebron Research Institute, Barcelona, Spain

## Introduction

Soluble guanylate cyclase (sGC), the main target of nitric oxide (NO), has been proven to have a significant role in coronary artery disease, pulmonary hypertension, erectile dysfunction and myocardial infarction. Several drugs that increase the activity of this enzyme are now in clinical phase of development: some of them are heme-dependent and might interact with the catalytic domain and others are heme-independent and supposedly bind to the sensory domain. The absence of reliable structural information is one of the factors that have precluded knowledge of the precise site of interaction of these molecules and of the mechanism of activation of the enzyme.

## Methods

Homology models of the catalytic domain of sGC in 'inactive' or 'active' conformation were constructed using, for the β-chain, the structure of recently published crystal of a nonphysiological homodimer of β subunits of human guanylate cyclase (2WZ1), for the α-chain, a similar domain of the green algae Chlamydomonas reinhardtii (3ET6) and, for monomer arrangement, the sGC 'inactive' structure (3ET6) or the 'active' catalytic domain of adenylate cyclase (1CJU). Molecular dynamics simulations of about 1μs each where run on all relevant models (NAMD/ACEMD, Amber99SB).

## Results

In the different trajectories, sGC conformation varied between having 1CJU- and 3ET6-like structures. One of these trajectories maintained extremely stable relative positions of the aminoacids in the catalytic site, being very similar to those described in 1CJU. The observed conformational transitions suggest a possible mechanism for the transmission of the cooperativity signal between the pseudo-symmetric and the catalytic site, in which Arg-92 (α-chain) and Arg-539 (β-chain) and the loop β2-β3 seem to play a critical role.

## Conclusions

Docking of YC-1, a classic heme-dependent activator, to all frames of this trajectory and absolute binding free energies with the linear interaction energy method (LIE) for selected poses revealed one potential binding site located between pseudo-symmetric and catalytic sites just over the loop β2-β3. This site would be compatible with the binding of a second GTP or an inhibitory ATP to the pseudo-symmetric site.

